# Rule-Based Detection of Structural Outliers in Non-Stationary Time Series

**DOI:** 10.3390/e28070724

**Published:** 2026-06-24

**Authors:** Marcin Kacprowicz

**Affiliations:** Institute of Information Technology, Lodz University of Technology, Al. Politechniki 8, 93-590 Lodz, Poland; marcin.kacprowicz@p.lodz.pl

**Keywords:** time series analysis, structural outliers, IF–THEN rules, deterministic algorithms, financial data analysis

## Abstract

Outlier detection in time series is traditionally formulated as the identification of rare or extreme observations with respect to global statistical properties. While effective for stationary processes, this perspective becomes insufficient in complex and non-stationary systems, where atypical behavior may manifest as disruptions of stable relationships rather than numerical extremeness. This paper proposes a rule-based framework for detecting structural outliers in non-stationary time series. Regular system behavior is represented by an interpretable set of deterministic IF–THEN rules describing stable relational patterns between features. Each rule defines a logical context and an admissible range of a diagnostic quantity, estimated nonparametrically from historical observations satisfying the rule condition. For a given observation, the set of active rules is identified and a structural inconsistency score is computed as the fraction of violated rule consequences. Additionally, observations lacking support from high-frequency contexts are treated as candidates for structural atypicality. The method is deterministic and avoids the need for explicit probabilistic modeling or iterative parameter learning, which simplifies interpretation and implementation. The framework is illustrated on daily EUR/USD data (2010–2022) using technical indicators (EMA, RSI) and absolute log-returns as the diagnostic measure. Results provide evidence that structurally atypical events can be identified even when global statistical thresholds remain unviolated, suggesting the practical relevance of relational analysis for non-stationary time series monitoring contexts.

## 1. Introduction

Information Systems (ISs) deployed in operational environments continuously generate time series data reflecting system behavior under evolving conditions. Monitoring such data streams for anomalous behavior is a recurring requirement across the information systems development (ISD) lifecycle, arising in system validation, quality monitoring, and operational maintenance. In regulated or safety-critical domains, detection decisions must be interpretable and traceable to explicit structural conditions, properties that global statistical methods often fail to provide.

Detecting outliers in time series is one of the key issues in data analysis in complex systems. In the classical approach, outliers are defined as rare, extreme, or significantly deviating from the global statistical properties of the data [1,2]. This approach works well in simple, stationary processes but proves insufficient in systems characterized by nonlinearity, structural variability, and locally changing relationships between variables [3,4].

Complex systems such as financial markets, sensor networks, and biological processes generate time series in which significant events do not necessarily manifest as extreme values [5,6]. In many cases, observations with “normal” numerical values may simultaneously violate the regular structural relationships that apply in the system. Such behaviors often remain undetected by methods based solely on global statistical measures.

In response to these limitations, this paper proposes interpreting outliers as structural violations of system regularities rather than numerical deviations from the distribution. Regular behavior is modeled using deterministic IF–THEN rules that provide interpretable, traceable descriptions of stable operational patterns, a property directly relevant to auditability requirements in IS monitoring and governance. The aim is to formulate a rule-based framework for detecting structural outliers and to evaluate it on real financial and synthetic data.

Problem statement.

Formally, given a time series of feature vectors ${\left\{x_{t}\right\}}_{t=1}^{n}$ and a set of structural rules R, the objective is to identify a subset O⊆{1,…,n} such that each t∈O corresponds to an observation that violates the stable relational structure encoded by R, as measured by the inconsistency score $V(x_{t})$ or by the absence of support from high-frequency rule contexts.

Contributions.

The main contributions of this work are as follows:

- A formal definition of *structural outlier* as an observation that violates stable relational patterns captured by IF–THEN rules, together with a clear distinction from regime changes and global statistical extremes.
- A deterministic inconsistency score $V(x_{t})$ that aggregates binary rule violations across overlapping contexts into a single interpretable measure, together with a no-context signal that identifies observations falling outside the structural coverage of the rule library. The key novelty is not the use of IF–THEN rules per se, but the combination of: (i) deterministic violation aggregation across multiple overlapping rule activations, (ii) explicit treatment of coverage gaps as a separate detection signal, and (iii) instance-level structural explanations absent from density-based methods.
- An out-of-sample empirical evaluation on real financial data and on a controlled synthetic system, demonstrating that the method detects structurally inconsistent observations that are invisible to global baselines.

The proposed framework is modular by design: rule sets may be defined globally as in this work, or extended with context-conditional activation and adaptive local updating, identified as natural directions for future research.

## 2. Related Work

The problem of outlier detection in time series has been widely studied from different methodological perspectives. Classical statistical approaches define outliers as observations that deviate significantly from a global model of the data, such as an assumed probability distribution or a fitted parametric process [1,2]. These methods are effective for stationary series but lose applicability when the underlying dynamics change over time [3]. A comprehensive survey of deep learning approaches to time series anomaly detection, covering both univariate and multivariate settings, is provided by Darban et al. [7].

A comprehensive taxonomy of outlier detection techniques for time series has been proposed by Blázquez-García et al. [8], who distinguish between point, subsequence, and whole-series outliers and classify methods by input type, detection technique, and the nature of the reference of normality. Their review highlights that most existing approaches rely on distributional or distance-based criteria and do not explicitly model structural relationships between variables.

Local density-based methods, such as the Local Outlier Factor (LOF) [9], define outliers relative to the density of their neighborhood in feature space. Isolation Forest [10] takes a complementary approach, isolating outliers through random recursive partitioning. While these methods can capture contextual deviations, they rely on distance or density measures rather than explicit structural relationships, and do not provide interpretable explanations for why a given observation is considered outlying.

A separate line of research addresses non-stationarity through regime segmentation. Hamilton [11] proposed Markov-switching models in which the dynamics of the system alternate between a finite number of states with different statistical properties. Truong et al. [12] provide a comprehensive review of change-point detection methods. These approaches focus on identifying regime boundaries rather than detecting individual outlying observations within or across regimes. A broader treatment of non-stationarity as concept drift—where the relation between input features and system behavior changes over time—is surveyed by Lu et al. [13], providing a general framework within which the structural variability addressed in this paper can be situated.

Schmidl et al. [14] conducted a large-scale empirical evaluation of over 70 time series outlier detection algorithms, revealing that no single method dominated across all settings and that interpretability remained a challenge for most high-performing approaches. Lai et al. [15] revisited the definitions and benchmarks used in time series outlier detection, arguing that ambiguous outlier definitions and inconsistent evaluation protocols limited the comparability of existing methods.

Rule-based approaches to data analysis offer an alternative that combines interpretability with structural expressiveness. Teste et al. [16] proposed human-interpretable rules for detecting unusual patterns in time series, and Nowak-Brzezińska [17] explored the identification of outliers within IF–THEN rule-based knowledge bases. The foundational framework for rule learning has been established by Fürnkranz et al. [18]. However, in most existing rule-based works, rules are defined globally and do not account for the contextual variability characteristic of non-stationary systems. The importance of inherently interpretable models in high-stakes decision environments—as opposed to post hoc explanations of black-box models—was argued forcefully by Rudin [19], providing additional motivation for the rule-based design adopted in this work.

The approach proposed in this paper differs from the above in several respects. First, it models regular system behavior using deterministic IF–THEN rules whose consequences are estimated nonparametrically within specific structural contexts, rather than from global distributions. Second, it formalizes outliers explicitly as violations of stable structural relationships rather than distributional extremes. Third, it introduces a deterministic inconsistency measure $V(x_{t})$ that aggregates binary rule violations across multiple overlapping contexts into a single interpretable score. Fourth, it integrates absence of rule support as a separate detection signal, distinct from rule violation. Each detection decision is accompanied by an instance-level explanation identifying the specific violated rules and their associated structural context.

Table 1 summarizes the key distinctions between the proposed method and representative existing approaches.

## 3. Structural Outlier Definition

From a structural perspective, it is crucial to distinguish between global normality and relational normality [20,21]. On this basis, we propose the following working definition:

*An outlier is an observation that violates the stable structural relationships of the system, even if its numerical values remain consistent with the global statistical properties of the data.*

In this paper, a violation of structural relationships is treated as a violation of the acceptable range of variability of the diagnostic quantity $y_{t}$, determined conditionally in relation to the active rule context. This definition shifts the emphasis from value analysis to relationship analysis.

**Distinction from regime change.** A *structural outlier* is a single observation (or a short sequence) that violates the stable relational patterns captured by the active rule set, while the system otherwise continues to operate within the same structural regime. A *regime change*, by contrast, refers to a persistent transition affecting a sustained segment. A regime change may initially manifest as a cluster of structural outliers, but the defining distinction is *persistence*. The proposed framework does not attempt to model regime changes directly; however, the frequency of detected structural outliers may serve as an indirect indicator of an ongoing regime transition.

## 4. Rule-Based Model of Regular System Behavior

The regular behavior of a complex system is described by a set of local structural relationships in the form of IF–THEN rules:

$$\mathrm{IF}\;C_{r}\left(x_{t}\right)\;\mathrm{THEN}\;y_{t}\in \left[L_{r},U_{r}\right],$$

where $C_{r}\left(x_{t}\right)$ defines the structural context, and $\left[L_{r},U_{r}\right]$ specifies the acceptable range of the diagnostic quantity $y_{t}$.

Each rule is characterized by its *support*, the frequency of activation of its context. Additional quality dimensions (consistency, persistence) are relevant but not formally operationalized in this baseline variant.

**Rule specification.** The rules are specified a priori based on established domain knowledge about financial time series [5,22]. The three rule contexts were chosen to capture the most fundamental structural dimensions of daily FX dynamics: trend structure (via the EMA ordering, which is the standard indicator of trend persistence), momentum regime (via RSI, the most widely used oscillator with conventional thresholds 30/70 proposed by [22]), and a trend-momentum interaction (correction within an uptrend). These indicators and thresholds are not arbitrary but follow standard practice in both academic and practitioner literature.

The deliberate use of a small, manually defined rule set isolates the detection mechanism from automatic rule generation, allowing the structural detection framework to be evaluated independently. The framework itself is agnostic to how rules are obtained; in practice, rules can be learned automatically using established rule-induction algorithms [18]. The small number of rules (three) is a limitation of this configuration, not of the framework.

**Support.** For each rule *r*, support is defined as $\sup\left(r\right)=\frac{1}{n}\sum_{t=1}^{n}1\left\{C_{r}\left(x_{t}\right)=\mathrm{true}\right\}$, where *n* is the number of observations in the training set. Rules with high support (sup(r)≥α) form the set $R^{high}$.

## 5. Detection of Outliers as Rule Violations

For observation $x_{t}$, the set of active rules is $R_{t}=\left\{r\in R:C_{r}\left(x_{t}\right)=\mathrm{true}\right\}$. The diagnostic quantity is $y_{t}=\left|\ln\left({\mathrm{Close}}_{t}/{\mathrm{Close}}_{t-1}\right)\right|$, where ${\mathrm{Close}}_{t}$ denotes the daily closing price at time *t*, and the consequence range $\left[L_{r},U_{r}\right]$ is estimated as $Q_{0.05}$ and $Q_{0.95}$ from training data satisfying $C_{r}$. The rule-consequence intervals $\left[L_{r},U_{r}\right]$ are estimated exclusively from the training data (2010–2018) and remain fixed during evaluation.

The binary violation function is:

$$v_{r}\left(x_{t}\right)=\left\{\begin{array}{cc} 1, & \mathrm{if}\;C_{r}\left(x_{t}\right)\wedge \left(y_{t}<L_{r}\vee y_{t}>U_{r}\right),\\ 0, & \mathrm{otherwise}. \end{array}\right.$$

Let $R_{t}^{high}=R_{t}\cap R^{high}$ denote the set of active high-support rules for observation $x_{t}$. The inconsistency score is:

$$V\left(x_{t}\right)=\left\{\begin{array}{cc} 1, & \mathrm{if}\;R_{t}^{high}=\emptyset ,\\ \frac{1}{|R_{t}^{high}|}\sum_{r\in R_{t}^{high}}v_{r}\left(x_{t}\right), & \mathrm{otherwise}. \end{array}\right.$$

**Detection criterion.** Observation $x_{t}$ is an outlier if $R_{t}\cap R^{high}=\emptyset$ or $V(x_{t})\geq \theta$. The no-context criterion reflects the assumption that observations outside well-supported contexts represent structural novelty or model incompleteness, both relevant in exploratory analysis. This design treats the model as a structural reference, and observations outside its coverage are considered informative rather than ignored. In the EUR/USD evaluation, this accounts for 16.4% of detections at θ=1.0.

The detection procedure is summarized in Algorithm 1.
**Algorithm 1** Local rule-based outlier detection.**Require:** Rules R, thresholds θ,α∈(0,1) **Ensure:** Outlier set O  1:  O←∅  2:  **for** each $x_{t}$, t=1,…,n **do**  3:         Compute $y_{t}$; identify $R_{t}$; let $R_{t}^{high}\leftarrow R_{t}\cap R^{high}$  4:         **if** $R_{t}^{high}=\emptyset$ **then** $V(x_{t})\leftarrow 1$
  5:         **else** $V\left(x_{t}\right)\leftarrow \frac{1}{|R_{t}^{high}|}\sum_{r\in R_{t}^{high}}v_{r}\left(x_{t}\right)$  6:         **end if**
  7:         **if** $V(x_{t})\geq \theta$ **then** $O\leftarrow O\cup \{x_{t}\}$  8:         **end if**
  9:  **end for**
10:  **return** O


The complexity is O(n·|R|), linear in *n* for fixed |R|.

## 6. Experiments

### 6.1. EUR/USD Case Study

Daily EUR/USD exchange rate data were obtained from the public Stooq database. (https://stooq.com/q/d/?s=eurusd, accessed on 10 February 2026). The dataset contains daily Open, High, Low, and Close (OHLC) prices from January 2010 to December 2022 (3366 trading days). Data were split chronologically: training 2010–2018 ($n_{\mathrm{train}}=2330$), test 2019–2022 ($n_{\mathrm{test}}=1037$). Features: Close, EMA (20), EMA (50), EMA (100), RSI (14). Three rule contexts were used: $C_{1}$: EMA20 > EMA50 > EMA100 (uptrend), $C_{2}$: 30 < RSI < 70 (neutral momentum), $C_{3}$: Close > EMA50 ∧ RSI < 40 (correction). With α=0.05, rules $C_{1}$ (sup = 0.344) and $C_{2}$ (sup = 0.903) belonged to $R^{high}$; $C_{3}$ (sup = 0.001) was excluded. Under LOYO-CV, the mean number of calibration observations satisfying $C_{1}$ was 1069 (range: 983–1158 across folds) and for $C_{2}$, it was 2805 (range: 2783–2840), providing ample sample sizes for reliable nonparametric estimation of $Q_{0.05}$ and $Q_{0.95}$. Rule $C_{3}$ was activated by at most four observations per calibration fold and was excluded from the detection criterion; it contributed zero detections in all reported results.

To quantify the estimation uncertainty of the rule-consequence bounds, bootstrap confidence intervals (B=2000 resamples) were computed for $Q_{0.05}$ and $Q_{0.95}$ across all 13 LOYO-CV folds. Table 2 reports the fold-averaged estimates and 95% confidence intervals. The intervals are narrow relative to the point estimates: for $C_{1}$, the mean CI width for $U=Q_{0.95}$ is 0.00139 (15.2% of the point estimate); for $C_{2}$, it is 0.00091 (8.5%). This confirms that the sample sizes available for calibration are sufficient for stable nonparametric quantile estimation.

The support values reported above were computed from the full training period (2010–2018) for illustration. In all experimental evaluations, however, parameters were re-estimated under the leave-one-year-out (LOYO) cross-validation protocol described in the next subsection, ensuring strict out-of-sample separation. The fixed training/test split (2010–2018/2019–2022) was not used as the primary evaluation protocol; LOYO-CV was used throughout.

### 6.2. Validation Protocol

To avoid in-sample evaluation, rule-consequence ranges $\left[L_{r},U_{r}\right]$ and support values sup(r) were estimated using a *leave-one-year-out* (LOYO) cross-validation protocol: for each year y∈{2010,…,2022}, all parameters were calibrated from the remaining 12 years, and detection scores were computed on year *y* using exclusively out-of-sample estimates. Results were aggregated over all 13 folds (N=3366 total observations). This ensured a strict separation between calibration and evaluation. Table 3 reports the resulting detection counts for each year.

The variation in detection rates across years reflects genuine changes in EUR/USD structural dynamics: years 2010, 2014–2015 show elevated rates coinciding with documented market stress periods (Euro sovereign debt crisis, SNB unpeg event), while 2013 and 2017 show the lowest rates, consistent with periods of relatively stable trending behavior.

### 6.3. Baselines

We compared against five methods: global *z*-score (3σ), IQR (k=1.5) [2], global $P_{95}$, LOF (k=20) [9], and Isolation Forest [10]. To assess the effect of feature space dimensionality, LOF and IF were evaluated in two configurations: (i) on the one-dimensional diagnostic variable $y_{t}$ only (LOF/IF 1D), enabling direct comparison with univariate baselines; and (ii) on the full feature vector $(y_{t},{\mathrm{EMA}}_{20/50}\;\mathrm{ratio},{\mathrm{EMA}}_{50/100}\;\mathrm{ratio},$ price-EMA deviation,RSI) (LOF/IF full), providing a fair multivariate comparison. All parameters were estimated from calibration data under the LOYO-CV protocol.

### 6.4. Synthetic Experiment

A two-regime system (regime *A*: $\sigma_{A}=0.003$; regime *B*: $\sigma_{B}=0.012$) with $N_{\mathrm{train}}=1000$, $N_{\mathrm{test}}=500$, $N_{\mathrm{out}}=10$ injected outliers (five contextual, five global). This setup simulated a scenario where the combined distribution masked regime-specific deviations. It was repeated over R=20 realizations.

To assess the statistical significance of the observed differences, two-sided Wilcoxon signed-rank tests were applied to the Recall scores across R=20 independent realizations. The proposed method achieved significantly higher recall than all univariate baselines reported in Table 4—*z*-score, IQR, $P_{95}$, LOF-1D, and IF-1D—(*p* < 0.0001 in each case). The difference in recall between the proposed method and LOF (full) was not statistically significant (p=0.157), consistent with the near-identical Rec.(Ctx) values (1.000 vs. 0.990±0.045). In contrast, IF (full) achieved substantially lower contextual recall (Rec.(Ctx) =0.160±0.179), indicating that not all multivariate anomaly detectors benefited equally from regime-informative features. The proposed method thus achieves superior or comparable contextual recall relative to all baselines, with the additional advantages of interpretability and O(n) complexity. Table 4 reveals an important nuance. When applied to the 1D variable $y_{t}$ only, LOF and IF achieve zero recall on contextual outliers—identical to simple global thresholds—because pooling both regimes masks the regime-specific deviation. However, when applied to the full feature space (including structural indicators correlated with regime), LOF achieves Rec.(Ctx) =0.990±0.045, nearly matching the proposed method. This result is important and should not be obscured: multivariate density-based methods can detect contextual outliers when provided with regime-informative features.

The practical distinction lies elsewhere. First, the proposed method achieves this performance without requiring density estimation or distance computation, with O(n) complexity versus $O(n^{2})$ for LOF. Second, and more importantly for IS applications, the proposed method provides a structural explanation for each detection: which rule was violated, under which context, and why. LOF and IF provide anomaly scores without interpretable justification. Third, the proposed method’s no-context signal (238 detections in LOYO-CV) identifies a class of observations not explicitly represented by density-based methods as structural coverage gaps—those falling outside the structural coverage of the rule library.

### 6.5. Out-of-Sample EUR/USD Results

Table 5 reveals three distinct detection regimes. Global threshold methods (*z*-score, IQR, $P_{95}$) are conservative (2–5%) and their detections are a subset of the proposed method’s detections, confirming that structural conditioning captures a strictly broader class of observations than univariate thresholding. LOF and Isolation Forest applied to the full feature space $\left(y_{t},\mathrm{EMA}\;\mathrm{ratios},\;\mathrm{RSI}\right)$ flag 18–26% of observations with moderate overlap (34–51%) with the proposed method, indicating that multivariate density-based methods capture a partially overlapping but distinct set of anomalies. The low overlap reflects the fundamental difference in detection philosophy: density-based methods flag observations globally rare in feature space, while the proposed method flags observations that violate structural relationships within their active rule context. Of 568 detections, 330 are rule violations and 238 are no-context cases.

### 6.6. Sensitivity Analysis

Table 6 reports detection counts under LOYO-CV for varying threshold θ.

Table 7 extends the sensitivity analysis to the choice of quantile bounds used for estimating $\left[L_{r},U_{r}\right]$. Three scenarios are reported: the baseline Q[5,95] used throughout the paper, a more conservative Q[2.5,97.5], and a stringent Q[1,99]. More conservative, wider bounds reduce the number of rule violations flagged (OV component), while the no-context component (OU) remains unchanged as it depends only on rule coverage, not on quantile bounds.

Table 8 reports detection counts under a complementary formulation based on empirical tail scores. For each observation $x_{t}$ with active high-support rules, an empirical tail score is computed for each active rule $r\in R_{t}^{high}$:

$$s_{t,r}=\min\;\left\{{\hat{F}}_{r}\left(y_{t}\right),\;1-{\hat{F}}_{r}\left(y_{t}\right)\right\},$$

where ${\hat{F}}_{r}$ denotes the empirical CDF of the calibration observations satisfying $C_{r}$. The uncorrected scenario ($y_{t}\notin \left[Q_{0.05},Q_{0.95}\right]$) reproduces the baseline OV/OU detection counts from Table 7. Four correction scenarios are reported. *Per-observation* corrections (Bonferroni with threshold $0.05/m_{t}$ and Benjamini–Yekutieli’s FDR [23] with q=0.05, where $m_{t}\leq 2$ is the number of active rules for observation $x_{t}$) treat the family of tests as the set of active rule evaluations contributing to a single observation-level decision. *Global* corrections apply Bonferroni and BY-FDR across all m=4197 active rule evaluations simultaneously; this represents the most conservative possible scenario and results in near-complete suppression of rule-violation detections (OV =1), reflecting the stringency of correcting for a large number of highly correlated evaluations. The no-context component (OU =238) is invariant across all scenarios.

The detection count is insensitive to θ in the range $\left[\frac{1}{3},1\right]$, because the discrete scale of $V(x_{t})$ with two active rules yields values in $\left\{0,\frac{1}{2},1\right\}$. The no-context branch (V=1 by convention) dominates the lower-threshold regime. This stability is a practical advantage: the threshold does not require careful tuning.

### 6.7. Ablation: Contribution of the No-Context Signal

The detection criterion has two components: rule violation ($V(x_{t})\geq \theta$ with active rules) and absence of high-support context ($R_{t}\cap R^{high}=\emptyset$). Table 9 quantifies their individual contributions under LOYO-CV.

The two components are mutually exclusive by construction: an observation either has active high-support rules (enabling violation scoring) or does not. The no-context signal contributes 238 detections (41.9% of all flagged observations) that would be entirely missed by rule-violation scoring alone. These correspond to observations falling outside the structural coverage of the rule library—a distinct type of structural anomaly reflecting model incompleteness or genuine structural novelty.

### 6.8. Interpretability

Table 10 presents four representative detections together with their rule-level explanations. Observations $t_{2}$ and $t_{3}$ show structural violations (unusually low $y_{t}$ within context) invisible to all baselines. The rule-based explanation provides specific, verifiable justification absent from global methods.

### 6.9. Qualitative Comparison

Table 11 summarizes the key properties of each method. The proposed method is the only approach that combines structural conditioning, interpretable rule-level explanations, determinism, and linear complexity. LOF and IF on the full feature space achieve indirect structure-awareness through regime-informative features but do not provide explicit structural explanations for individual detections.

## 7. Discussion

The proposed method detects outliers as local structural violations, focusing on relationships between variables rather than global distribution properties. Global threshold baselines (*z*-score, IQR, $P_{95}$) flag only 2–5% of observations, and their detections are fully contained within the proposed method’s detection set. Multivariate LOF and IF flag 18–26% with moderate overlap (34–51%), indicating that density-based methods operating on the full feature space capture a partially overlapping but distinct anomaly class. These results suggest that structural rule-based conditioning and multivariate density estimation are complementary rather than competing approaches.

The LOYO-CV results (Table 3) reveal meaningful variation in detection rates across years, with elevated rates in 2010, 2014–2015 and 2022 coinciding with documented periods of EUR/USD structural stress. The lowest rates in 2013 and 2017 correspond to periods of relatively stable trending behavior. This temporal pattern provides qualitative evidence that the method captures genuine structural dynamics rather than noise.

The ablation study (Table 9) establishes that the no-context signal contributes 41.9% of all detections under LOYO-CV. It is important to note that this signal should not be interpreted as unambiguous evidence of anomaly. An observation falling outside the structural coverage of the rule library ($R_{t}\cap R^{high}=\emptyset$) may reflect either genuine structural novelty or a gap in model coverage. In IS monitoring contexts, both interpretations are operationally relevant: the former signals a potential anomaly, the latter signals that the rule library requires extension. The proposed framework treats both cases as warranting human review, which is appropriate in regulated environments where ignoring out-of-coverage observations carries risk.

From the perspective of information systems development, the interpretability of the proposed method is a key practical advantage. Each flagged observation is accompanied by an instance-level explanation identifying the specific violated rules and their associated context (Table 10). This traceability satisfies auditability requirements common in regulated IS environments and supports human-in-the-loop validation of detection decisions, a property absent from density-based and isolation-based methods.

The proposed framework is deterministic and non-parametric: it does not perform iterative model fitting and has no parameters optimized on a held-out validation set. The evaluation protocol used throughout—leave-one-year-out cross-validation (LOYO-CV)—provides strict out-of-sample separation between calibration and evaluation, analogous to the role of a test set in supervised learning. A three-way training/validation/test split is not applicable here because there are no hyperparameters selected by minimizing a validation loss; the two scalar parameters θ and α are set to default values whose insensitivity is demonstrated in Table 6. The use of chronological rather than random splits is deliberate and methodologically required: random assignment of years would introduce temporal leakage, allowing future distributional information to contaminate historical calibration.

The complementary empirical tail score analysis in Table 8 further reports per-observation and global Bonferroni and Benjamini–Yekutieli’s FDR variants. These results make the effect of multiple-comparison-style corrections explicit: per-observation corrections reduce rule-violation detections moderately (from 330 to 275–276), whereas global correction across all 4197 active rule evaluations simultaneously is extremely conservative and suppresses nearly all rule-violation detections (OV =1), leaving only the no-context component.

The inconsistency score $V(x_{t})$ is a deterministic aggregation of binary rule violations, not a statistical test. It does not involve hypothesis testing, *p*-values, or a null distribution, and therefore multiple-testing corrections such as Bonferroni or Benjamini–Yekutieli’s FDR control [23] are not directly applicable: there is no family of null hypotheses over which Type I error accumulates. Nevertheless, the sensitivity of detection counts to the stringency of the quantile bounds is a practically relevant question. Table 7 addresses this by reporting results under three bound scenarios. Moving from the baseline Q[5,95] to the conservative Q[2.5,97.5] reduces rule-violation detections from 330 to 173 (−47.6%); the stringent Q[1,99] reduces them further to 71. The no-context component (238 detections) is invariant to this choice. These results show that the detection counts are sensitive to the conservatism of the bounds in a predictable and monotone fashion, and that even under the most stringent scenario the method retains meaningful detection coverage (9.2% of observations).

The method requires two parameters: the violation threshold θ and the support threshold α. As shown in Table 6, detection counts are insensitive to θ in the range $\left[\frac{1}{3},1\right]$ due to the discrete structure of $V(x_{t})$ with two active rules. The support threshold α=0.05 ensures that only rules activated by at least 5% of training observations contribute to detection, providing a minimum sample size of approximately 117 observations for reliable percentile estimation. In practice, both parameters can be set to default values (θ=1, α=0.05) without significant performance sensitivity.

The method’s determinism and O(n) complexity make it practical for large-scale IS deployments. The main limitation is the manual design of rule contexts. With only three rules (two with high support), the inconsistency score $V(x_{t})$ takes values in {0,0.5,1.0}, limiting discrimination between moderate and severe violations. Expanding the rule library would increase the granularity of $V(x_{t})$ and improve the method’s ability to distinguish partial from complete structural inconsistency. Preliminary experiments with an expanded eight-rule set (adding downtrend, overbought, oversold, and short-term trend contexts) showed that $|O_{U}|$ dropped to zero—all observations were covered by at least one high-support rule— and the detection count became meaningfully sensitive to θ, confirming that rule library size is the primary lever for controlling detection behavior.

The sensitivity of results to the specific choice of rules (e.g., RSI thresholds 25/75 vs. 30/70, or EMA windows 10/30/90 vs. 20/50/100) has not been exhaustively analyzed in this work. The sensitivity analysis in Table 6 addresses the effect of θ but not of individual rule parameterization, which is a direction for future research.

Future work includes automatic rule induction, context-conditional rule activation with adaptive local updating, and application to non-financial IS monitoring domains.

## 8. Conclusions

This paper proposed a rule-based framework for detecting structural outliers in non-stationary time series, in which unusual observations are interpreted as violations of stable relational patterns rather than global statistical deviations. The method uses deterministic IF–THEN rules with nonparametrically estimated consequences and identifies outliers either by rule violation or by absence of structural coverage, two complementary signals shown via ablation to contribute independently to detection performance.

Out-of-sample evaluation under a leave-one-year-out cross-validation protocol on EUR/USD data (2010–2022) confirmed that the method detects structurally inconsistent observations, including cases not flagged by global baselines and only partially overlapping with LOF and Isolation Forest detections. The controlled synthetic experiment demonstrated perfect recall on contextual outliers—observations that are structurally atypical within their regime but globally unremarkable—while all univariate baselines achieved zero recall on this outlier type. Multivariate LOF on the full feature space achieved comparable recall on contextual outliers, though without the interpretability and O(n) complexity of the proposed method.

The method retains full interpretability, O(n) complexity, and resistance to non-stationarity, making it directly applicable to IS monitoring and operational data analysis in regulated environments. Future work will extend the approach with context-conditional rule activation, automatic rule induction, and adaptive updating for evolving system dynamics.

## Figures and Tables

**Table 1:** Comparison of outlier detection approaches along key methodological dimensions.

Property	Proposed	Global Stat.	LOF/IF	Regime Switching	Rule-Based
Structural relationships	Yes	No	No	Partial	Yes
Deterministic	Yes	Yes	No	No	Yes
Interpretable explanation	Yes	No	No	No	Partial
No-support detection	Yes	No	No	No	No
Non-parametric	Yes	No	Yes	No	Partial
O(n) complexity	Yes	Yes	No	No	Yes

**Table 2:** Bootstrap 95% percentile confidence intervals for rule-consequence bounds $L=Q_{0.05}$ and $U=Q_{0.95}$. Intervals were computed separately within each LOYO-CV calibration fold using B=2000 resamples; the table reports mean point estimates and mean lower/upper CI limits across the 13 folds. CI width is reported as absolute value.

Rule	*n* (Mean)	*L* (Mean)	95% CI for *L*	*U* (Mean)	95% CI for *U*	CI Width for *U*
$C_{1}$	1069	0.000270	[0.000215,0.000323]	0.009126	[0.008563,0.009950]	0.00139
$C_{2}$	2805	0.000277	[0.000232,0.000315]	0.010640	[0.010185,0.011094]	0.00091

**Table 3:** LOYO-CV detection counts per year (θ=1.0, $N_{\mathrm{year}}\approx 259$).

Year	Outliers	%	No-Context
2010	86	33.2	42
2011	51	19.6	4
2012	38	14.6	23
2013	29	11.2	0
2014	75	29.0	40
2015	76	29.3	38
2016	31	12.0	15
2017	16	6.2	0
2018	31	12.0	19
2019	19	7.4	2
2020	27	10.4	16
2021	41	15.8	18
2022	48	18.6	21
**Total**	**568**	**16.9**	**238**

**Table 4:** Synthetic detection performance (mean ± std, R=20). Ctx = contextual outliers, Glob = global outliers.

Method	Prec.	Recall	$F_{1}$	Rec.(Ctx)	Rec.(Glob)
Proposed (rules)	0.177±0.019	1.000±0.000	0.300±0.028	1.000±0.000	1.000±0.000
*z*-score (3σ)	0.364±0.075	0.500±0.000	0.417±0.050	0.000±0.000	1.000±0.000
IQR (k=1.5)	0.137±0.031	0.500±0.000	0.213±0.035	0.000±0.000	1.000±0.000
$P_{95}$	0.170±0.030	0.500±0.000	0.252±0.032	0.000±0.000	1.000±0.000
LOF (1D)	0.323±0.099	0.500±0.000	0.385±0.065	0.000±0.000	1.000±0.000
IF (1D)	0.321±0.063	0.500±0.000	0.388±0.046	0.000±0.000	1.000±0.000
LOF (full)	0.504±0.086	0.995±0.022	0.665±0.073	0.990±0.045	1.000±0.000
IF (full)	0.337±0.072	0.580±0.089	0.422±0.068	0.160±0.179	1.000±0.000

**Table 5:** Out-of-sample detections and overlap (EUR/USD, LOYO-CV, N=3366, θ=1.0).

Method	Det.	%	Overlap w. Prop.	Excl. Baseline
Proposed (θ=1.0)	568	16.9	—	—
LOF (k=20, full)	864	25.7	295 (34.1%)	569
IF (full)	618	18.4	314 (50.8%)	304
LOF (k=20, 1D)	677	20.1	177 (26.1%)	500
IF (1D)	590	17.5	360 (61.0%)	230
*z*-score (3σ)	71	2.1	71 (100%)	0
IQR (k=1.5)	136	4.0	136 (100%)	0
$P_{95}$	172	5.1	172 (100%)	0

**Table 6:** Detection counts under LOYO-CV as a function of threshold θ (N=3366).

θ	Detections	%
$\frac{1}{3}$	589	17.5
$\frac{1}{2}$	589	17.5
$\frac{2}{3}$	568	16.9
1	568	16.9

**Table 7:** Detection counts under LOYO-CV for conservative quantile bound variants (N=3366, θ=1.0). OV = rule violations; OU = no-context detections.

Quantile Bounds	OV	OU	Total	%
Q[5,95] (baseline)	330	238	568	16.9
Q[2.5,97.5] (conservative)	173	238	411	12.2
Q[1,99] (stringent)	71	238	309	9.2

**Table 8:** Detection counts under LOYO-CV for empirical tail score decision scenarios (N=3366, θ=1.0). OV = rule violations; OU = no-context detections (invariant). $m_{t}\leq 2$ active rules per observation; m=4197 total active rule evaluations across all observations.

Level	Scenario	OV	OU	Total	%
Per-obs.	Uncorrected ($y_{t}\notin \left[Q_{0.05},Q_{0.95}\right]$)	330	238	568	16.9
Per-obs.	Bonferroni ($s_{t,r}\leq 0.05/m_{t}$)	275	238	513	15.2
Per-obs.	BY-FDR (q=0.05)	276	238	514	15.3
Global	Bonferroni ($s_{t,r}\leq 0.05/m$)	1	238	239	7.1
Global	BY-FDR (q=0.05, m=4197)	1	238	239	7.1

**Table 9:** Ablation study under LOYO-CV (N=3366, θ=1.0).

Criterion	Detections	%
Full criterion (union)	568	16.9
Rule violation only	330	9.8
No-context only	238	7.1
Both simultaneously	0	0.0

**Table 10:** Example detections with rule-level explanations (θ=1.0).

*t*	$y_{t}$	RSI	Active	Violated	*z*	IQR	$P_{95}$
$t_{1}$	0.0108	38.5	$C_{2}$	$C_{2}$: $y_{t}>U_{2}$	No	No	Yes
$t_{2}$	0.0002	59.6	$C_{2}$	$C_{2}$: $y_{t}<L_{2}$	No	No	No
$t_{3}$	0.0001	63.4	$C_{1},C_{2}$	Both: $y_{t}<L_{r}$	No	No	No
$t_{4}$	0.0133	68.5	$C_{1},C_{2}$	Both: $y_{t}>U_{r}$	No	Yes	Yes

**Table 11:** Qualitative comparison of methods.

Property	Proposed	*z*-Score	IQR	$P_{95}$	LOF	IF
Contextual sensitivity	Yes	No	No	No	No	No
Interpretable explanation	Yes	No	No	No	No	No
Structure-aware	Yes	No	No	No	No *	No *
Distribution-free	Yes	No	Yes	Yes	Yes	Yes
Deterministic	Yes	Yes	Yes	Yes	No	No
Complexity	O(n)	O(n)	O(n)	O(n)	$O(n^{2})$	O(nlogn)

* LOF and IF on the full feature space achieve structure-awareness indirectly via regime-informative features rather than through explicit structural conditioning; see Table 4 (Synthetic detection performance) for the corresponding empirical results.

## Data Availability

The data analyzed in this study are publicly available from Stooq at https://stooq.com/q/d/?s=eurusd (accessed on 10 February 2026).
